# Younger Age and Parenchyma-Sparing Surgery Positively Affected Long-Term Health-Related Quality of Life after Surgery for Pancreatic Neuroendocrine Neoplasms

**DOI:** 10.3390/jcm12206529

**Published:** 2023-10-14

**Authors:** Anna Caterina Milanetto, Claudia Armellin, Gloria Brigiari, Giulia Lorenzoni, Claudio Pasquali

**Affiliations:** 1 Chirurgia Generale 3, Pancreatic and Digestive Endocrine Surgery Research Group, Department of Surgery, Oncology and Gastroenterology, University of Padova, 35128 Padova, Italy; 2 Unit of Biostatistics, Epidemiology and Public Health, Department of Cardiac, Thoracic, Vascular Sciences and Public Health, University of Padova, 35128 Padova, Italy

**Keywords:** pancreas, neuroendocrine neoplasm, health-related quality of life, EORTC questionnaire, pancreatic surgery, parenchyma-sparing surgery

## Abstract

(1) Background: Patients with pancreatic Neuroendocrine Neoplasms (PanNENs) often have a long overall survival. We evaluated determinants of quality of life (QoL) after surgery for PanNENs. (2) Methods: Patients operated on for a PanNEN in our center (1990–2021) received three EORTC QoL questionnaires (QLQ-C30, QLQ-GI.NET21, QLQ-PAN26). Six domains were selected as outcome variables (global QoL, physical function -PF, social function -SF, disease-related worries -DRWs, pain, upper-gastrointestinal (GI) symptoms) and evaluated in relation to the clinical variables. Statistical analysis was performed using R software v 4.2.2. (3) Results: One hundred and four patients enrolled showed a good global QoL (median 83.3). Old age was a determinant of worse global QoL (*p* 0.006) and worse PF (*p* 0.003). Multiple comorbidities (*p* 0.002) and old age (*p* 0.034) were associated with pain, while male gender was related to better PF (*p* 0.007) and less pain (*p* 0.012). Patients who had undergone parenchyma-sparing surgery demonstrated better PF (*p* 0.037), better SF (*p* 0.012), and less upper-GI symptoms (*p* 0.047). At multivariable analysis, age (*p* 0.005) and type of surgery (*p* 0.028) were confirmed as determinants of global QoL. (4) Conclusions: In patients operated on for a PanNEN, a good HRQoL is generally reported; notably, younger age and parenchyma-sparing surgery seem to positively affect HRQoL.

## 1. Introduction

Neuroendocrine neoplasms of the pancreas (PanNENs) are a heterogeneous group of neoplasms arising from cells in the diffuse neuroendocrine system located in the islets of Langerhans [1]. According to the Surveillance Epidemiology and End Results (SEERs) program, their incidence is currently estimated at 0.7 per 100,000, with a slight increase during the last years [2]. While a part of PanNENs produce biologically active peptides and are usually associated with distinct clinical syndromes (Functioning, F-PanNENs), most PanNENs are not related to a clinically significant hormone hyperproduction and are referred to as Non-Functioning (NF-PanNENs) [3]. According to their histopathological features, PanNENs are divided into well-differentiated neuroendocrine tumors (NETs) and poorly differentiated neuroendocrine carcinomas (NECs), with the latter having the worst prognosis and thus avoiding surgical resection. Surgery is the only curative treatment for PanNENs and is the best choice when tumors are localized to the pancreas, varying from enucleation to major pancreatic resections.

Patients with a PanNET usually show a favorable prognosis once operated on and a long survival when compared to other abdominal neoplasms [2], even if presenting with distant metastases at diagnosis. In the case of metastatic disease, other treatments may be required: loco-regional treatment (i.e., trans-arterial embolization for liver metastases) and systemic treatments, such as somatostatin analogues (SSAs), peptide receptor radionuclide therapy (PRRT), targeted therapy (i.e., Everolimus, Sunitinib), and chemotherapy, depending on tumor differentiation and grade.

Health-related quality of life (HRQoL) is an outcome of paramount importance in PanNEN patients, and it is a multidimensional construct that needs to be assessed as a patient-reported outcome [4]. The European Organization for Research and Treatment of Cancer (EORTC) is a leading association in determining HRQoL, and it constructed several questionnaires to assess that outcome in different neoplastic settings. The quality of life of NEN patients has been investigated in several recent studies [5,6,7,8,9,10,11,12,13], mentioning the impact of medical treatments in HRQoL, and using EORTC questionnaires as reference tools. Caplin et al. [5] in a study about the effect of SSAs on metastatic gastro-entero-pancreatic (GEP)-NETs demonstrated comparable levels of global QoL between the treatment and the placebo groups; Jimenez-Fonseca [6] confirmed the clinical benefit of SSA and also sunitinib, clearly supported by HRQoL assessment. Pavel et al. [7] reported that everolimus was associated with a worsening of HRQoL for GEP-NET patients, and Ramage et al. [8] showed that HRQoL is maintained in patients with PanNENs during treatment with everolimus, even if disease progression or death were recorded in 44% of patients during follow-up. An improvement in global QoL was reported after PRRT by Teunissen et al. [9] and Marinova et al. [10] in terms of increasing global health and the mitigation of physical complaints, whereas Martini et al. [11] showed in GEP-NET patients an overall stable HRQoL under PRRT but significant HRQoL impairments compared with the general population. Finally, in a recent systematic review and metanalysis by Ronde et al. [12], all treatments considered for GEP-NENs (SSA, PRRT, targeted therapies, and chemotherapy) appeared beneficial for disease stabilization while maintaining stable global health status, even if high-quality HRQoL reporting was lacking.

Most of the published studies about HRQoL in NEN patients involved not only pancreatic NENs but GEP-NEN patients globally, as it was also reported in a recent systematic review by Watson et al. [13], that a heterogeneous group of NENs consists of neoplasms arising from different organs which show different biological behavior and prognosis, thus the patients’ outcomes are not comparable. To the best of our knowledge, this is the first study reporting about HRQoL determinants only in surgically treated PanNENs.

The primary endpoint of our study was to investigate HRQoL in patients who had already undergone a previous surgery for a PanNEN using the EORTC QoL questionnaires, and to verify if patients operated on for a PanNEN have an HRQoL of more than 66/100 in the functional scale and less than 33/100 in the symptoms scale. A secondary endpoint was to evaluate determinants of HRQoL, and to verify if patient-related (i.e., age, gender, other diseases, postoperative pancreatic function), disease-related (i.e., tumor grade and symptoms, tumor burden), or treatment-related (i.e., type of surgery, SSAs, systemic treatments) factors may influence the HRQoL. Another secondary endpoint was to compare the HRQoL of PanNEN patients with the HRQoL of the general population and of all cancer and hepato-pancreato-biliary (HPB) cancer populations, as reported in the EORTC reference values manual, and to verify if patients operated on for a PanNEN have a better HRQoL than the people affected by all/HPB cancer, and a slightly worse HRQoL than the general population.

## 2. Materials and Methods

### 2.1. Study Population

Patients who underwent surgery for a PanNEN between 1st January 1990 and 31st December 2021 in our Pancreatic Surgical Unit were identified from hospital computerized systems. Inclusion criteria were histologically confirmed diagnosis of PanNEN, and at least six months of follow-up after surgery. The majority of patients were enrolled during a routinely outpatient clinic follow-up, while the others were contacted by phone, were sent the questionnaires, and they returned the completed consent forms and questionnaires by mail. Clinical records of the included patients were retrieved (gender, age, other diseases, MEN-1 diagnosis, tumor functional state, tumor grade, type of surgery, tumor burden, SSA therapy, systemic/loco-regional treatment, further pancreatic surgery, and pancreatic function) and entered into an anonymized database. Written informed consent was obtained from all the patients. The study was approved by the local Ethical Committee (reference number 5091/AO/21). Enrollment of patients, questionnaire administration, and clinical data collection were carried out from 1 January 2022 to 31 December 2022.

### 2.2. HRQoL Assessment

To assess HRQoL in PanNEN patients, we used the Italian version of three different questionnaires from the EORTC website: EORTC QLQ-C30, EORTC QLQ-GI.NET21, and EORTC QLQ-PAN26 [14]. The EORTC Core Quality of Life Questionnaire (EORTC QLQ-C30) is a generic questionnaire which measures cancer patients’ physical, psychological, and social functions, and it consists of 30 items divided into 16 functional scales, 12 symptom scales, and 2 global health and QoL items [15]. The EORTC gastro-intestinal NET questionnaire (EORTC QLQ-GINET21) is a specific questionnaire for gastro-intestinal (GI) NEN patients, and it includes 21 items (10 functional and 11 symptom scales) [16,17], and the EORTC pancreatic cancer questionnaire (EORTC QLQ-PAN26) is a specific questionnaire for pancreatic cancer patients, and it consists of 26 items (11 functional and 15 symptom scales) [18]. The scores for each item were first standardized to a 0–100 linear scale according to the EORTC QLQ Scoring Manual [19]. For the functional scales and global QoL, a high score represents a high level of functioning (i.e., better HRQoL), whereas for the symptom scales, a high score indicates a high level of symptomatology (i.e., worse HRQoL).

The three different questionnaires may include similar domains of HRQoL; for example, the domain “social functioning”, is called “SF” in QLQ-C30, “SF21” in QLQ-GINET21, and “SF26” in QLQ-PAN26. Therefore, we created global domains consisting of all the items describing the same functioning or symptom scale among the three questionnaires (Table 1).

Finally, we selected the domains to be considered for the subsequent data analyses according to the current literature and to their relevance in the specific field of PanNENs. The following HRQoL domains were considered as outcome variables and evaluated in relation to the clinical predictive variables: global QoL, physical functioning (PF), social functioning (SF), disease-related worries (DRWs), pain (PA, including pancreatic and bone–muscle pain), and upper-gastrointestinal symptoms (upper-GI, including appetite, nausea/vomiting, abdominal discomfort, acid ingestion/heartburn, difficulties with eating, restrictions in type and quantity of food, indigestion, itching, and jaundice).

Every chosen domain was first elaborated on separately, and a weighted average of the scores of each item grouped in each domain was calculated for every patient. Missing data were managed following indications reported in the EORTC QLQ-C30 Scoring Manual [19]: if at least half of the items of every single domain reported a valid score, the average score was considered valid. We considered as “missing data” all the missed answers due to a non-pertinent question (expressed as “NA” in the questionnaires, i.e., questions regarding systemic treatments’ side effects for patients who did not receive any of those treatments) and all the missed answers due to a non-given answer. Median values (with interquartile ranges) were calculated.

Clinical predictive variables were considered binary whenever possible, as follows: gender (male vs. female), age (65–90 years vs. 20–64 years), MEN1 diagnosis (yes vs. no), PanNEN type (non-functioning vs. functioning), tumor grade (G2 vs. G1), surgery (parenchyma-sparing vs. standard resection), other treatments after surgery (SSA therapy, systemic/locoregional treatment, and/or redo surgery: yes vs. no), and postoperative pancreatic function (normal vs. exocrine insufficiency and/or new-onset diabetes). In the other cases, we established cut-off values as follows: comorbidities (no vs. single vs. multiple), and tumor burden (no evidence of disease vs. local recurrence vs. distant metastases).

### 2.3. Statistical Analysis

Descriptive statistics were reported as median (I–III quartiles) for continuous variables and percentages (absolute numbers) for categorical variables. The association between baseline characteristics and QoL scores was evaluated using the univariable Gamma model to account for the non-normal distribution of the outcomes. The marginal effect was computed considering the partial derivatives of the marginal expectation. Results were reported as average marginal effect (AME), 95% CI, and *p*-value. The AME should be interpreted as the mean change in the outcome variables (functional or symptom scales) per unit increase in the independent variable. Analyses were performed with R software v 4.2.2 [20] together with the package margins.

## 3. Results

In total, 112 patients operated on for a PanNEN in our Pancreatic Surgical Unit between 1990 and 2021 met the inclusion criteria, and 104 patients provided all the questionnaires needed and were enrolled in the study (response rate 92.9%). The median time from surgery to questionnaire completion was 109 (range, 6–384) months. Clinical data of the study population are reported in Table 2.

There were 41 men and 63 women, with a mean age of 63 (SD 13.5) years. Among them, 58% had non-functioning PanNEN (75% G1), 64% had multiple comorbidities, 54% had undergone a standard pancreatic resection, and 83% of patients showed a normal (or not impaired) pancreatic function at the time of the study. As reported in Table 3, patients showed good global QoL results (median 83.3; IQR 58.3–100) and reported low DRWs (median 26.7; IQR 13.3–33.3). Physical (median 94.4; IQR 77.8–100) and social (median 88.9; IQR 77.8–94.4) functions were modestly affected. Pain (median 9.2; IQR 0–19.1) and upper-GI symptoms (median 3.9; IQR 0–9.1) were rarely reported.

**Figure 1:**
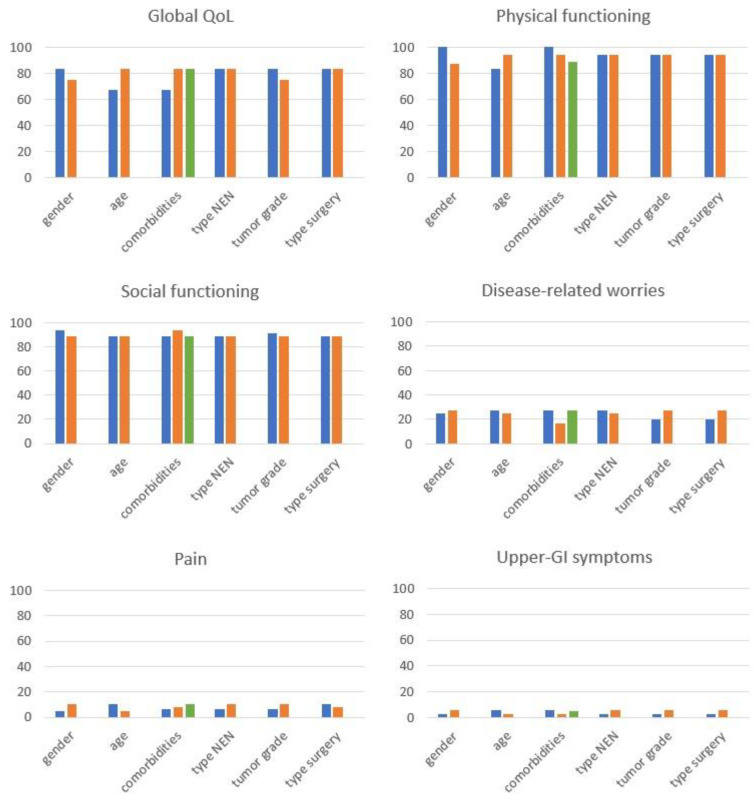
Distribution of EORTC scores according to the variables of interest. Data are median. **Legend**. Gender (male, in blue vs. female, in orange), age (65–90 years, in blue vs. 20–64 years, in orange), comorbidities (no, in blue vs. single, in orange vs. multiple, in green), type of pancreatic neuroendocrine neoplasm (non-functioning, in blue vs. functioning, in orange), tumor grade (G2, in blue vs. G1, in orange), surgery (parenchyma-sparing, in blue vs. standard resection, in orange). QoL, quality of life. GI, gastrointestinal.

Distribution of EORTC scores according to the variables of interest are reported in Figure 1 and also detailed in the Supplementary Materials. Then, in Table 4, we have detailed the results of the univariable analysis.

First, a worse global QoL was found to be significantly associated with older age (AME = −0.12; 95% CI = −0.21, −0.03; *p* value 0.006). No association was detected between global QoL and gender, comorbidities, type of NEN, tumor grade, or type of surgery.

Among the functional scales considered, a worse PF was found to be significantly associated with older age (AME −0.12; 95% CI −0.2, −0.04; *p* 0.003), whereas a significantly better PF was observed in male patients (AME 0.11; 95% CI 0.03, 0.19; *p* 0.007) and in patients who had undergone parenchyma-sparing surgery (AME 0.09; 95% CI 0.01, 0.17; *p* 0.037). Parenchyma-sparing surgery was also associated with a better SF (AME 0.09; 95% CI 0.02, 0.16; *p* 0.012).

In the analysis of symptomatic scales, pain was less complained by male patients (AME −0.08; 95% CI −0.14, −0.02; *p* 0.012), while it scored worse results both in older age (AME 0.07; 95% CI 0.01, 0.14; *p* 0.034) and in the case of multiple comorbidities (AME 0.10; 95% CI 0.04, 0.15; *p* 0.002). The presence of a single comorbidity was not related to significant changes in pain; this was also independent from tumor type, tumor grade, and type of surgery. Type of surgery showed a correlation with upper-GI symptoms, with better results in case of parenchyma-sparing surgery (AME −0.04; 95% CI −0.08, 0, *p* 0.047). Finally, DRWs showed no statistically significant correlations at all.

Within the multivariable analysis, three clinical variables were finally chosen in relation to global QoL depending on their theoretical meaningfulness for the study of NENs and on the significance of univariable analysis results: age, gender, and type of surgery. Data confirmed the significance of age (AME −0.12; 95% CI −0.22, −0.04; *p* 0.005) and type of surgery (AME 0.10; 95% CI 0.01, 0.19; *p* 0.028) in the determination of global QoL, while gender was not substantially related to QoL in this population (AME 0.07; 95% CI −0.02, 0.17; *p* 0.12).

## 4. Discussion

In this observational single-center study, 104 patients showed a good global QoL (median 83.3; IQR 58.3–100) after pancreatic surgery for a PanNEN. Despite the need for a pancreatic resection, a correct diagnosis and several treatment options may provide an excellent quality of life. The major long-term HRQoL determinants for those patients were found to be gender, age, and type of surgery, with age and surgery confirmed also as determinants of global QoL at multivariable analysis.

The male gender was related to a better PF (AME 0.11; *p* 0.007), and to a higher tolerance for pain (AME −0.08; *p* 0.012). In a similar study regarding HRQoL in patients operated on for a small intestinal NEN [21], the female gender and old age were found to be associated with worse outcomes. Then, treatment with SSAs and non-symptomatic NENs were associated with a better QoL, but those statistically significant results could not be verified in the present study due to biological and clinical differences existing between pancreatic and small intestinal NENs, and thus in the variables investigated.

In our study, men showed modestly increased scores for social relations and globally lower rates of DRWs; however, when comparing men and women, no significant implications can be suggested in the relation and attitude of both genders toward their neoplasm. In a recent study, Pijnappel et al. [22] investigated the experience of fear of tumor recurrence or progression in patients with pancreatic cancer treated with surgical resection, palliative systemic treatment, or best supportive care. In that study, and also according to our results, even if overall survival was not directly related to QoL, patients need to be guided by healthcare professionals through their treatment journey to deal with the internal distress caused by the fear of disease.

A globally worse QoL may be reasonably assumed in elderly people, and lower scores were also confirmed in our study, where at the time of questionnaire completion, PanNEN patients had a mean age of 63 years. Notably, 75% of patients underwent surgery for a grade 1 PanNET, a histological feature harboring a low risk of tumor recurrence, and patients’ long survival. Old age was a determinant of a worse global QoL, confirmed at multivariable analysis (AME −0.13; *p* 0.005). In a recent study, Okuyama et al. [23] showed a general deterioration of the activities of daily living (ADL) among older patients after surgery for cancers of the GI and HPB tracts. However, the heterogeneity of the tumors included in that study does not make any comparison made with our results reliable.

As might be expected, in the present study, old age played a crucial role in the impairment of PF (AME −0.12; *p* 0.003), with a progressively increasing incidence of chronic diseases (multiple comorbidities in 64% of patients), and thus of soreness and pain complaints, as confirmed by the significant correlation between pain and both old age (AME 0.07; *p* 0.034) and multiple comorbidities (AME 0.1; *p* 0.002). A recent longitudinal study by Modica et al. [24] analyzed HRQoL in 39 patients with a GEP-NEN, with about half of them diagnosed with a PanNEN. HRQoL was assessed in relation to clinical severity and heterogeneity of NENs, as well as resilience. A higher number of therapies and lower levels of resilience were associated with lower global QoL scores and higher levels of symptomatic scales, while patients with a GEP-NEN showed higher HRQoL scores in many HRQoL domains; then, no statistically significant differences were highlighted between patients who underwent surgery and those who did not. Unfortunately, the global data reported for all GEP-NENs patients made a comparison with the present study unfeasible.

In our study, 54% of patients underwent a standard pancreatic resection, and 83% maintained normal (or not impaired) pancreatic function after surgery. Concerning parenchyma-sparing resections for PanNEN and benign neoplasms, the reported incidence of new onset/worsening diabetes mellitus and of exocrine insufficiency is 1–8% and 2–8%, respectively [25,26]. In our population, parenchyma-sparing resections were related to better PF (AME 0.09; *p* 0.037) and SF (AME 0.09; *p* 0.012), and they ensured a lower risk of developing upper-GI symptoms (AME −0.04; *p* 0.047), and the HRQoL domain that no other tumor nor patients’ features seemed to affect. Short- and long-term outcomes, including QoL, were also assessed in a recent series of 81 patients surgically treated by pancreatic enucleation [27]; despite significant postoperative morbidity rates, those patients reported excellent long-term outcomes and a QoL being comparable to the general population. 

The EORTC QLQ-C30 Manual [28] includes reference values for QLQ-C30, and it has a specific section for QoL in the general population and in different groups of cancer patients. Notably, in that manual, an HPB cancer patients’ group is analyzed, consisting mainly of men aged more than 50 years and of 298 pancreatic cancer patients (out of 750 total HPB cancer patients). Those patients showed a scarce global QoL (median value 58.3, IQR 41.7–75.0) with good functional results (both PF and SF) but high rates regarding fatigue, insomnia, and appetite loss symptoms [28]. All cancer patients globally reported a discrete global QoL (median value 66.7, IQR 50.0–83.3), with functional scales median values over 80.0, and complaining above all about fatigue, pain, and insomnia (median values more than 25.0) [28]. Our study population of surgically treated PanNEN patients showed excellent outcomes (median global QoL over 83.0 and median values of functional and symptoms scales ranging between 80.0 and 100 and 4.0 and 27.0, respectively) when compared to HPB and all cancer patients. Moreover, PanNEN patients’ outcomes are similar to the results reported in the EORTC manual for the general population, with a median global QoL of 75.0 (IQR 58.3–83.3), functional scales with a median of 100 in both PF and SF, and symptomatic scales’ rates globally less than 25.0 [27].

Our study has some strengths and limitations.

Regarding the strengths, this study reports a single-center case series of high numerosity when considering that PanNENs represent a rare disease. This is the first study reporting about HRQoL determinants only in patients who were surgically treated for a PanNEN. All enrolled patients were operated on and followed-up at the same pancreatic surgical center, so treatment choices appear homogeneous over the years. Patients were followed-up regularly in the postoperative period and were examined for HRQoL after a long-term follow-up, with a median time of nine years after surgery. Finally, among the patients in regular follow-up first asked to join the study, we observed a very high response rate (93%) to the questionnaires.

Regarding the limitations, the research planning started in 2020, and we decided to administer to pancreatic NEN patients the three validated EORTC questionnaires, which could better investigate HRQoL in that peculiar study population. In fact, specific EORTC questionnaires for functioning and/or NF-PanNENs were not available at that time. Patients made a great effort to answer all the questions of the three questionnaires, which sometimes were not applicable to their personal experience, and we also had to merge all the items of similar domains among the different questionnaires to ensure a proper data analysis with reliable results. In the spring of 2023, EORTC released two new questionnaires regarding PanNETs (QLQ-P.NET15 for gastrinoma/nonfunctioning and QLQ-PNET19 for insulinoma) which are still in the validation phase.

## 5. Conclusions

This is the first study evaluating long-term HRQoL after surgery in patients with a PanNEN. In this subset of patients, younger age and parenchyma-sparing surgery seem to positively affect HRQoL, and a good global QoL is reported even when compared to the general population. Elderly women who have undergone a standard pancreatic resection and are affected by multiple comorbidities show the worst HRQoL outcomes and may be the subset of operated PanNEN patients who need more support by the healthcare system and healthcare professionals.

## Figures and Tables

**Table 1:** Domain creation (all items included). In blue, items from EORTC-QLQ-C30. In yellow, items from EORTC-PAN26. In orange, items from EORTC-GiNET21. QoL, quality of life. PF, physical functioning. EF, emotional functioning. SX, sexuality. SF, social functioning. RF, role functioning. HCS, healthcare satisfaction. CF, cognitive functioning. PA, pain. TR, treatment-related symptoms. LGI, lower gastrointestinal symptoms. OS, other symptoms. UGI, upper-gastrointestinal symptoms. FI, financial difficulties. ED, endocrine symptoms. DRWs, disease-related worries. BI, body image.

**Global QoL**															
29	30														
**Functional Scales**															
PF									EF				SX		
1	2		3			4	5	42	21	22	23	24	55	56	51
SF									RF		HCS			CF	
26	27		42			44	49	52	6	7	53	54	50	20	25
**Symptom Scales**															
PA										TR					
9	19		31			33	34	35	48	38	43	50	39	40	
LGI											OS				
16	17		35			36	32	40	46	47	8	10	11	12	18
UGI														FI	
13	14		15			34	37	38	36	37	39	44	45	28	
ED						DRWs					BI				
31	32		33			41	43	47	41	51	48	49	45	46	

**Table 2:** Clinical features of the study population (n = 104).

At the Time of Surgery		
Gender, n (%)	Male	41 (39.4)
	Female	63 (60.6)
Type of NEN, n (%)	Functioning ^1^	44 (42.3)
	Nonfunctioning	60 (57.7)
Tumor grade, n (%)	G1	73 (75.3)
	G2	24 (24.7)
	N/A	7
Type of surgery, n (%)	Parenchyma-sparing resection ^2^	48 (46.1)
	Standard resection ^3^	56 (53.9)
MEN-1 syndrome, n (%)	Yes	18 (17.3)
	No	86 (82.7)
**At the time of the study**		
**Age (years)**	median (range)	63 (20–90)
	Under 65	53 (51.0)
	More/equal 65	51 (49.0)
**Comorbidities, n (%)**	No	13 (12.6)
	Single	24 (23.3)
	Multiple	66 (64.1)
	N/A	1
**Tumor burden, n (%)**	NED	91 (87.5)
	LR	6 (5.8)
	DM	7 (6.7)
**Systemic and/or locoregional treatments, n (%)**	Current	5 (4.8)
	Previous	2 (1.9)
	No	97 (93.3)
**SSA therapy, n (%)**	Current	10 (9.6)
	Previous	2 (1.9)
	No	92 (88.5)
**New surgery, n (%)**	Yes ^4^	6 (5.8)
	No	98 (94.2)
**Pancreatic function, n (%)**	New onset diabetes mellitus	5 (4.8)
	Exocrine insufficiency	5 (4.8)
	Exocrine/Endocrine insufficiency	8 (7.7)
	Normal	86 (82.7)

**Legend**. NEN, neuroendocrine neoplasm. MEN, multiple endocrine neoplasia. SS-A, somatostatin analogues. N/A, missing data. NED, no evidence of disease. LR, local recurrence. DM, distant metastases. ^1^ In total, 34 insulinoma, 7 gastrinoma, 2 VIPoma, 1 glucagonoma. ^2^ In total, 33 enucleation, 11 central pancreatectomy, 4 duodenum-preserving pancreatic head resection. ^3^ In total, 1 total pancreatectomy, 9 pancreato-duodenectomy, 34 distal pancreatectomy, 12 spleen-preserving distal pancreatectomy. All results are reported as number of patients (apart from age). ^4^ In MEN1 patients.

**Table 3:** Preliminary analysis of questionnaires’ data.

Outcome Variable (Missing Data) ^1^	Median Value	*IQR*	Min	Max
**Global QoL**	83.3	58.3–100	5.6	100
**Functional scales**				
Physical functioning (0.2%)	94.4	*77.8–100*	5.6	100
Role functioning (0.5%)	100	*83.3–100*	0.0	100
Emotional functioning (1.2%)	91.7	*75.0–100*	25.0	100
Cognitive functioning	100	*83.3–100*	16.7	100
Social functioning (2.9%)	88.9	*77.8–94.4*	27.8	100
Healthcare satisfaction (8.3%)	77.8	*66.7–100*	0.0	100
Sexuality (23.7%)	100	*100–100*	0.0	100
**Symptomatic scales**				
Disease-related worries (10.2%)	26.7	*13.3–33.3*	8.0	80.0
Body image (8.4%)	8.3	*0.0–16.7*	0.0	66.7
Financial difficulties (1%)	0.0	*0.0–0.0*	0.0	100
Pain (1%)	9.2	*0.0–19.0*	0.0	80.9
Endocrine symptoms (1.3%)	0.0	*0.0–16.7*	0.0	88.9
Treatment-related symptoms (35.6%)	0.0	*0.0–11.1*	0.0	44.4
Lower-GI symptoms (0.4%)	8.3	*4.2–20.8*	0.0	62.5
Upper-GI symptoms (1%)	3.9	*0.0–9.1*	0.0	66.7
Other symptoms ^2^ (0.2%)	13.3	*0.0–26.7*	0.0	73.3

**Legend**. QoL, quality of life. GI, gastrointestinal. IQR, interquartile range. ^1^ The percentage of missing data is reported in brackets. ^2^ Fatigue, dyspnea, and sleep disorders.

**Table 4:** Results of the gamma regression. Data are reported as average marginal effect, 95% CI, and *p*-value.

	Functional Scales						Symptomatic Scales					
	Global QoL		Physical Functioning		Social Functioning		Disease-Related Worries		Pain		Upper-GI Symptoms	
	AME (95% CI)	*p* Value	AME (95% CI)	*p* Value	AME (95% CI)	*p* Value	AME (95% CI)	*p* Value	AME (95% CI)	*p* Value	AME (95% CI)	*p* Value
Gender												
Male	0.08 (−0.01, 0.17)	*0.082*	0.11 (0.03, 0.19)	***0.007***	0.05 (−0.02, 0.12)	*0.17*	−0.05 (−0.12, 0.03)	*0.211*	**−0.08** **(−0.14, −0.02)**	***0.012***	−0.02 (−0.06, 0.02)	*0.351*
Female												
Age												
>/=65 y	**−0.12** **(−0.21, −0.03)**	***0.006***	**−0.12** **(−0.2, −0.04)**	***0.003***	−0.06 (−0.13, 0.01)	*0.1*	0.05 (−0.02, 0.13)	*0.149*	**0.07** **(0.01, 0.14)**	***0.034***	0.04 (−0.01, 0.09)	*0.082*
<65 y												
**Comorbidities**												
No												
Single	0.06 (−0.1, 0.23)	*0.44*	−0.03 (−0.18, 0.12)	*0.712*	0.02 (−0.11, 0.15)	*0.792*	−0.05 (−0.16, 0.06)	*0.352*	0.03 (−0.03, 0.09)	*0.304*	−0.01 (−0.06, 0.04)	*0.607*
Multiple	−0.05 (−0.19, 0.09)	*0.499*	−0.11 (−0.24, 0.02)	*0.105*	−0.07 (−0.18, 0.04)	*0.236*	0.05 (−0.05, 0.16)	*0.331*	**0.1** **(0.04, 0.15)**	***0.002***	0.03 (−0.02, 0.08)	*0.223*
**Type of NEN**												
NF	−0.02 (−0.11, 0.07)	*0.718*	0 (−0.08, 0.08)	*0.989*	−0.03 (−0.1, 0.04)	*0.399*	0.03 (−0.04, 0.11)	*0.381*	−0.01 (−0.08, 0.06)	*0.737*	0.02 (−0.02, 0.06)	*0.346*
F												
**Tumor grade**												
G2	0.06 (−0.05, 0.17)	*0.267*	−0.01 (−0.11, 0.08)	*0.794*	0 (−0.08, 0.08)	*0.963*	−0.02 (−0.1, 0.66)	*0.65*	−0.03 (−0.11, 0.04)	*0.339*	−0.01 (−0.06, 0.03)	*0.61*
G1												
**Type of surgery**												
Limited	0.07 (−0.02, 0.16)	*0.141*	**0.09** **(0.01, 0.17)**	***0.037***	**0.09** **(0.02, 0.16)**	***0.012***	−0.06 (−0.13, 0.01)	*0.108*	−0.01 (−0.08, 0.05)	*0.693*	**−0.04** **(−0.08, 0)**	***0.047***
Standard												

**Legend**. QoL, quality of life. GI, gastrointestinal. AME, average marginal effect. NEN, neuroendocrine neoplasm. NF, nonfunctioning. F, functioning.

## Data Availability

The data presented in this study are available on request from the corresponding author. The data are not publicly available due to privacy.

## Supplementary Materials

The following supporting information can be downloaded at: https://www.mdpi.com/article/10.3390/jcm12206529/s1, Table S1. Distribution of EORTC scores according to the variables of interest. Data are median [I quartile-III quartile].
