# Supplementary material for: Younger Age and Parenchyma-Sparing Surgery Positively Affected Long-Term Health-Related Quality of Life after Surgery for Pancreatic Neuroendocrine Neoplasms
Source: J Clin Med. 2023 Oct 14;12(20):6529. doi: 10.3390/jcm12206529 (PMC10607516; doi:10.3390/jcm12206529)
Supplement: Supplementary file 1 [file jcm-12-06529-s001.zip › jcm-2653388-supplementary.pdf]

**Table S1.** Distribution of EORTC scores according to the variables of interest. Data are median [I quartile-III quartile].

| | Functional Scales | | | Symptomatic Scales | | |
|---|---|---|---|---|---|---|
| | Global QoL | Physical Functioning | Social Functioning | Disease-Related Worries | Pain | Upper-GI Symptoms |
| | Median (IQR) | Median (IQR) | Median (IQR) | Median (IQR) | Median (IQR) | Median (IQR) |
| **Gender** | | | | | | |
| Male | 0.83 (0.67-1.00) | 1.00 (0.94-1.00) | 0.94 (0.80-1.00) | 0.25 (0.08-0.33) | 0.05 (0.00-0.10) | 0.03 (0.00-0.09) |
| Female | 0.75 (0.58-0.88) | 0.87 (0.72-0.94) | 0.89 (0.78-0.94) | 0.27 (0.16-0.40) | 0.10 (0.05-0.23) | 0.06 (0.00-0.09) |
| **Age** | | | | | | |
| >/= 65 y | 0.67 (0.50-0.83) | 0.83 (0.67-0.94) | 0.89 (0.76-0.94) | 0.27 (0.17-0.40) | 0.10 (0.05-0.24) | 0.06 (0.03-0.15) |
| < 65 y | 0.83 (0.67-1.00) | 0.94 (0.89-1.00) | 0.89 (0.83-0.94) | 0.25 (0.08-0.33) | 0.05 (0.00-0.14) | 0.03 (0.00-0.08) |
| **Comorbidities** | | | | | | |
| No | 0.67 (0.67-1.00) | 1.00 (0.94-1.00) | 0.89 (0.83-0.94) | 0.27 (0.13-0.33) | 0.05 (0.00-0.10) | 0.06 (0.03-0.09) |
| Single | 0.83 (0.73-1.00) | 0.94 (0.83-1.00) | 0.94 (0.83-0.97) | 0.17 (0.07-0.29) | 0.08 (0.04-0.15) | 0.03 (0.00-0.06) |
| Multiple | 0.83 (0.58-0.92) | 0.89 (0.72-1.00) | 0.89 (0.72-0.94) | 0.27 (0.17-0.40) | 0.10 (0.00-0.24) | 0.05 (0.00-0.12) |
| **Type of NEN** | | | | | | |
| NF | 0.83 (0.58-1.00) | 0.94 (0.75-1.00) | 0.89 (0.78-0.94) | 0.27 (0.17-0.40) | 0.05 (0.00-0.19) | 0.03 (0.00-0.09) |
| F | 0.83 (0.67-1.00) | 0.94 (0.78-1.00) | 0.89 (0.82-0.94) | 0.25 (0.08-0.33) | 0.10 (0.05-0.19) | 0.06 (0.00-0.09) |
| **Tumor grade** | | | | | | |
| G2 | 0.83 (0.79-1.00) | 0.94 (0.88-1.00) | 0.91 (0.88-0.94) | 0.20 (0.13-0.37) | 0.05 (0.00-0.10) | 0.03 (0.00-0.06) |
| G1 | 0.75 (0.58-0.92) | 0.94 (0.78-1.00) | 0.89 (0.78-0.94) | 0.27 (0.13-0.33) | 0.10 (0.00-0.19) | 0.06 (0.00-0.09) |
| **Type of surgery** | | | | | | |
| Limited | 0.83 (0.67-1.00) | 0.94 (0.82-1.00) | 0.89 (0.83-1.00) | 0.20 (0.07-0.27) | 0.10 (0.00-0.19) | 0.03 (0.00-0.07) |
| Standard | 0.83 (0.58-0.92) | 0.94 (0.67-1.00) | 0.89 (0.72-0.94) | 0.27 (0.17-0.40) | 0.08 (0.00-0.19) | 0.06 (0.00-0.12) |

**Legend.** QoL, quality of life. GI, gastrointestinal. IQR, interquartile range. NEN, neuroendocrine neoplasm. NF, nonfunctioning. F, functioning.
